# The social income inequality, social integration and health status of internal migrants in China

**DOI:** 10.1186/s12939-017-0640-9

**Published:** 2017-08-04

**Authors:** Yanwei Lin, Qi Zhang, Wen Chen, Li Ling

**Affiliations:** 1 Faculty of Medical Statistics and Epidemiology, School of Public Health, Sun Yat-sen University, Guangzhou, China; 2 Center for Migrant Health Policy, Sun Yat-sen University, Guangzhou, China; 3 School of Public Health, Baotou Medical College, Baotou, China; 4 School of Community and Environmental Health, Old Dominion University, Norfolk, VA USA; 5 Present Address: Sun Yat-sen University, (North Campus), #74, Zhongshan Road II, Guangzhou, 510080 People’s Republic of China

**Keywords:** Social income inequality, Social integration, Health, China

## Abstract

**Background:**

To examine the interaction between social income inequality, social integration, and health status among internal migrants (IMs) who migrate between regions in China.

**Methods:**

We used the data from the 2014 Internal Migrant Dynamic Monitoring Survey in China, which sampled 15,999 IMs in eight cities in China. The Gini coefficient at the city level was calculated to measure social income inequality and was categorized into low (0.2 < Gini <= 0.3), medium (0.3 < Gini <= 0.4), high (0.4 < x < = 0.5), and very high (Gini >0.5). Health status was measured based upon self-reported health, subjective well-being, and perceptions of stress and mental health. Social integration was measured from four perspectives (acculturation and integration willingness, social insurance, economy, social communication). Linear mixed models were used to examine the interaction effects between health statuses, social integration, and the Gini coefficient.

**Results:**

Factors of social integration, such as economic integration and acculturation and integration willingness, were significantly related to health. Social income inequality had a negative relationship with the health status of IMs. For example, IMs in one city, Qingdao, with a medium income inequality level (Gini = 0.329), had the best health statuses and better social integration. On the other hand, IMs in another city, Shenzhen, who had a large income inequality (Gini = 0.447) were worst in health statues and had worse social integration.

**Conclusion:**

Policies or programs targeting IMs should support integration willingness, promote a sense of belonging, and improve economic equality. In the meantime, social activities to facilitate employment and create social trust should also be promoted. At the societal level, structural and policy changes are necessary to promote income equity to promote IMs’ general health status.

## Background

Internal migrants (IMs) are individuals who migrate between regions within one country [1]. Since the 1950s, the Chinese government has maintained a household registration (“Hukou”) system that defines access to employment, housing, social welfare, and educational opportunities in order to restrict the geographical mobility of the population [2]. But with economic development, the attitude of government toward internal mobility has shifted from restriction to assistance. For example, 15 cities with high concentrations of IMs were designated as “demonstration pilot cities” of IM social integration in 2014. According to the Report on China’s Migrant Population Development of 2015, the number of IMs reached 253 million in 2014. Like international migrants, IMs experience similar social inclusion challenges, since China has significant disparities in culture, economic development, and social environments across regions [3, 4].

Literature on international migrants and internal migrants suggests that migration has an impact on both physical and mental health [5–7]. The healthy migrant paradox has suggested that immigrants have physical health advantages at the initial stage of the immigration, but this advantage diminishes significantly with increasing residence time in the host society, a fact that was also observed in IMs in China, particularly among the temporary rural-to-urban migrant population [2, 8, 9]. Attempts to explain the decrease of the migrant physical health advantage have attributed the phenomenon to two factors: a temporal lifestyle that emphasizes immigrants’ assimilation to host society life and acculturative stress [10]. Meanwhile, more studies are concentrating on IMs’ mental health status. A few studies suggested that IMs suffered from stress arising from work-, family- and interpersonal-related difficulties and thus had higher levels of psychological distress than residents [11–14]. Possible contributing factors included economic status and cultural adaptation status [3]. However, most studies on IMs’ health factors have just focused on a particular aspect of social life, such as economic status or acculturation. [15–19]. Only a few studies have explored the IMs’ health status from a more comprehensive social perspective including personal and social factors.

One of the societal factors rarely examined in IMs is social income inequality, which could affect public health across societies [20, 21]. For example, Wilkinson and Pickett (2006) reviewed 168 studies and found more than 70% of them reported that health was worse in societies with greater income inequality [22]. In China, researchers have tended to discuss the influence of social income inequality from the health resources allocation’s perspective instead of from the perspective of the impact on health per se [23, 24]. In our study, we complement the literature by examining the association between social income inequality and the health statuses of IMs in eight cities with high IM concentrations in China.

Another social factor that might affect IMs’ health status is social integration. The concept of social integration was developed to understand and explain immigrants’ behavior, adaptation, the acculturation process, and self-identity. Social integration is a multi-dimensional concept that has multiple definitions across disciplines [25]. It is commonly measured by the size of the IM’s network, frequency of contact with network members, and membership in a formal or informal organization [26, 27]. In this study, we adopted a theoretical framework of social integration that uses economic status, social communication, acculturation, and self-identity [28].

The relationship between social integration and health status could be complicated to measure among immigrants due to lack of accurate measurement tools. Researchers have been prone to focus on a particular aspect of social integration. For example, in western countries, Butler (2015) found that individual psychological resources, social support, the acculturation process, cultural variations, and time since relocation were significant protective factors against the development of common mental disorders amongst migrants [29]. Haasen (2008) examined the association between acculturation stress and mental distress in migrants from Russia to Germany and found a significant correlation between acculturation stress and mental distress [30]. In China, Lei (2012) examined the relationship between migrants’ social ties and their mental health and discovered that more trans-local ties were associated with better mental health, whereas the number of local ties was not a significant health protector [31]. Wen (2010) found that neighborhood satisfaction, social cohesion, and safety showed a strong association with health after controlling for individual factors [32]. However, migration is a complicated process involving all aspects of society, so the various influences on health should be explored comprehensively.

In this study, we referred to a concept model proposed by Lisa F. Berkman in 2000 [33], which envisioned a cascading causal process beginning with the macro-social (social-structural conditions) to the mezzo-social (social integration) to personal health. We chose social income equality measured by the Gini coefficient to reflect socioeconomic disparities in the hosting society for IMs. We also evaluated IMs’ social integration with respect to economic status, social insurance, social communication, culture adoption, and identity. This study explored the relationship between social integration and health status under varying degrees of socioeconomic inequality. Our objective is to complement the existing literature by providing further insights into the social factors that might influence the health statuses of IMs in China. Our results may help policy makers design the proper social policies to improve IMs’ social integration and health statuses.

## Method

### Study site and data collection

Data came from the Internal Migrant Dynamic Monitoring Survey, which was conducted by the National Population and Family Planning Commission in China in April 2014. The analysis of public access data was exempted by the local IRB; as this involved analyzing de-identified existing data, ethical approval was not required. The available data for this study included eight “demonstration pilot cities” of social integration (Chengdu, Jiaxing, Qingdao, Xiamen, Shenzhen, Beijing, Zhengzhou, Zhongshan). From the perspective of economic development and urban scale, Shenzhen and Beijing are first-tier cities, Chengdu, Zhengzhou, Qingdao, and Xiamen are second-tier cities, and Jiaxing and Zhongshan are third-tier cities. The sample size for each city was 2000, except for Chengdu having 1999, for a total sample size of 15,999.

### Measurement

#### The Gini coefficient

The Gini coefficient is a valid index measuring the extent of income inequality. The value of the Gini Coefficient varies from 0 (representing perfect income equality) to 1 (representing perfect income inequality). The currently accepted standard is: Gini ≤0.2 denotes absolute equality, 0.2 < Gini ≤0.3 means low inequality in our study, 0.3 < Gini ≤0.4 indicates medium inequality, and 0.4 < Gini ≤0.5 means high inequality, while values larger than 0.5 mean very high inequality [34]. In our study, all the Gini coefficients at the city level were between 0.2 to 0.5. We extracted the monthly income of IMs in the survey and divided them into 13 intervals (every 500RMB), then adopted the simple Gini coefficient calculation method to examine each city’s income equality [35].

#### Social integration

The variables selected to measure social integration were chosen based upon the indicator system proposed by Yang and Zhou [28, 36]. A total of 13 indicators were selected to indicate comprehensive integration in terms of socioeconomic status, social interaction, culture adoption, and integration willingness in the host city. The 13 indicators are: composition of the neighbors; familiarity with local dialects; planning to move family members to the migration city in the next 1–3 years; monthly household income; income or occupation position compared with the relatives, friends and colleagues of the current resident; degree of respect compared with relatives, friends and colleagues of the current resident; the consent of the views, integration willingness, discrimination perception, old-age insurance, medical insurance, number of organizations participated in, and number of activities attended (see Appendix 1 Table 5 for more details).

#### Health

Health status was measured from four angles: self-reported health, subjective well-being, perception of stress, and mental health. Self-reported health was appraised by the general health dimension of SF-36 (Cronbach’s coefficient = 0.72) that includes five items, with each item using a five point scale [37]. Subjective well-being was measured by the Satisfaction with Life Scale (SWLS, Cronbach’s coefficient = 0.73) that is a 5-item set of questions scored on a seven point scale from 1 (disagree strongly) to 7 (agree strongly) [38]. Perception of stress was evaluated by the 4-item perceived stress scale (PSS-4, Cronbach’s alpha coefficient = 0.79) [39]. Mental health was evaluated with the K6 scale of psychological well-being, with the Cronbach’s alpha coefficient as 0.80 [40]. In our study, higher values on the scales represent better self-reported health, subjective well-being, and mental health, but also more perceived stress.

#### Statistical methods

Stratified, multi-stage sampling was adopted based on the Probability Proportionate to Size Sampling method (PPS). All migrants who were reported by each village or neighborhood committee formed the basic sampling framework. Multilevel random selection was applied (townships were randomly selected from each city and then village, or neighborhoods were randomly selected from each selected township). Twenty individual subjects were extracted randomly in each selected village or neighborhood. Selected IMs were defined as individuals without “Hukou” (a registered resident certificate) in the place of residence who had been living in that location for more than a month. All sampled IMs were between 15 and 59 years of age.

Descriptive analyses were used to present demographic characteristics and health status. Analysis of variance (ANOVA) was used to test the difference of social integration and health statuses across cities. As for IMs’ social integration, the principal component factor analysis was adopted to extract common factors and to compute the scores. Out of the indexes, monthly household income was standardized based on the average family’s income in each city. Two-level (city and individual, the Gini coefficient for city-level units) linear mixed models were used to examine the associations between health statuses, social integration, and the Gini coefficient. Demographic characteristics, four factors of social integration, and the interaction effects of Gini and social integration were tested in fixed effect models.

## Result

### Demographic characteristics

The analytical sample included 15,999 IMs across eight cities. Adults aged 25 to 44 years old accounted for 68.1% of the sample. Most of them migrated from rural districts (86%), and 59.9% of them had received an education to the middle school level or below. The mean length of residence was 4.25 years (SD, 4.43), and the average age was 32.69 years old (SD, 8.72) (Table 1).

**Table 1:** Respondent’s Socio-demographic Characteristics in 2014 (*N* = 15,999)

Variable	Subgroup	n (%)
Gender	female	7200 (45.0)
	male	8799 (55.0)
Age (years)	15~	3661 (22.9)
	25~	6687 (41.8)
	35~	4212 (26.3)
	45~	1304 (8.2)
	55 ~ 59	135 (0.8)
Category of HuKou	agriculture	13,759 (86.0)
	non-agriculture	2240 (14)
Education	middle school and below	9590 (59.9)
	high school and above	6409 (40.1)
Marital status	single	4290 (26.8)
	married	11,709 (73.2)
Years of residence	<1 year	5248 (32.8)
	1 year~	2437 (15.2)
	2 years~	1481(9.3)
	3 years~	1369 (8.6)
	4 years~	1073 (6.7)
	5 years~	2803 (17.5)
	≥10 years	1588 (9.9)

### The Gini coefficient

The Gini coefficients were 0.278 and 0.289 respectively in Jiaxing and Chengdu, which indicates low income inequality. Zhengzhou (0.318), Qingdao (0.329), Xiamen (0.330), Zhongshan (0.336), and Beijing (0.375) were at medium income inequality levels. The Gini coefficient of Shenzhen was 0.447, which indicates high income inequality.

### Social integration

#### Factor analysis

We found that correlation coefficients between the variables were fit for principal component analysis (Kaiser-Meyer-Olkin Measure of Sampling Adequacy = 0.644). Communalities of variables were over 0.5, except the consent of the views (0.38), and therefore the principal component analysis was suitable for social integration of IMs. We chose the varimax method in factor rotation. Factors in which eigenvalues were greater than 1 were extracted, which produced 6 components and explained 65.32% of the total variance. The structure loading of factors extracted and component score coefficient matrix is presented in Appendix 2: Table 6.

Four factors were extracted according to loadings of the 6 components. The consent of the views (X6), integration will (X7), and discrimination perception (X8) constituted the first factor, called “acculturation and integration willingness.” The second factor was called “social insurance,” which was composed of old-age insurance (X9) and medical insurance (X10). The third factor was “subjective and objective economic status” as socioeconomic status (X4: Income or occupation position compared with the relatives, friends, and colleagues of the current resident; X5: Degree of respect compared with relatives, friends, and colleagues of the current resident; X13: Monthly household income; and X3: Bring family members or not to the local area in the next 1–3 years). The fourth factor, “social communication,” was constructed as a composition of neighbors (X1), familiarity with the local dialect (X2), number of organizations participated in (X11), and number of activities attended (X12) (Appendix 2: Table 6).

#### Social integration estimate

According to the result of factor analysis, scoring formulas of social integration were shown as below (Formula 1 to 5).

Factor 1 (acculturation and integration willingness, 14.56–62.44) = 0.526 * X6 + 0.789 * X7 + 0.812 * X8 (1).

Factor 2 (social insurance, 0–1.75) = 0.866 * X9 + 0.882 * X10 (2).

Factor 3 (socioeconomic status, 2.50–21.83) = 0.869 * X4 + 0.847 * X5 + 0.785 * X3 + 0.742 * X13 (3).

Factor 4 (social communication, 1.48–18.35) = 0.839 * X11 + 0.819 * X12 + 0.771 * X1 + 0.706 * X2 (4).

Social integration (2.65–16.26) = 0.126 Factor 1 + 0.119 Factor 2 + 0.206 Factor3 + 0.201 Factor 4 (5).

Beijing, the capital of China, was best in social insurance and economic status. Chengdu topped in social communication, acculturation, integration willingness, and social integration. Zhongshan was the worst in social insurance, and Zhengzhou was worst in subjective and objective economic status. Interestingly, Jiaxing, which has the highest income equality (Gini = 0.278), was lowest in social integration. Its scores of social communication, acculturation, and integration willingness were the lowest, too (Table 2). In general, social insurance ranked the first, with 74% of the total score of full integration, followed by acculturation and integration will (68%), socioeconomic status (54%), and social communication (24%). Social integration was 55% and improved over time.

**Table 2:** Estimated Value of Social Integration (Dimension Score and Comprehensive Score)

By Gini Coefficient	0.2 < Gini ≤0.3		0.3 < Gini ≤0.4					0.4 < Gini ≤0.5	
	Jiaxing	Chengdu	Zhengzhou	Qingdao	Xiamen	Zhongshan	Beijing	Shenzhen	Total
Acculturation and integration will ^***^	41.47 ± 3.87	43.90 ± 3.28	42.79 ± 3.51	43.52 ± 3.75	41.25 ± 4.1	41.84 ± 3.83	42.57 ± 3.63	42.53 ± 3.92	42.48 ± 3.85
Social insurance^***^	1.19 ± 0.64	1.44 ± 0.50	1.31 ± 0.49	1.35 ± 0.58	1.15 ± 0.68	1.06 ± 0.69	1.51 ± 0.42	1.37 ± 0.55	1.30 ± 0.60
Socioeconomic status ^***^	11.60 ± 2.51	11.67 ± 2.71	11.06 ± 2.5	12.36 ± 2.68	11.69 ± 3.17	11.96 ± 2.89	12.41 ± 2.78	11.82 ± 3.52	11.82 ± 2.89
Social communication^***^	3.18 ± 1.31	5.61 ± 1.76	5.27 ± 1.72	4.25 ± 1.25	3.58 ± 1.77	3.73 ± 1.49	4.70 ± 1.77	5.16 ± 1.57	4.43 ± 1.80
Social integration ^***^	8.42 ± 0.86	9.26 ± 0.87	8.91 ± 0.8	9.07 ± 0.87	8.49 ± 0.1	8.64 ± 0.9	9.07 ± 0.92	9.02 ± 1.05	8.86 ± 0.96
By Years of Resident	Years of Residence								
	<1 year	1 year~	2 years~	3 years~	4 years~	5 years~		≥10 years	
Acculturation and integration will ^***^	42.26 ± 3.83	42.29 ± 3.90	42.73 ± 3.64	42.67 ± 3.78	42.48 ± 4.01	42.64 ± 3.86		42.85 ± 3.86	
Social insurance^***^	1.24 ± 0.61	1.28 ± 0.61	1.36 ± 0.57	1.32 ± 0.59	1.33 ± 0.58	1.35 ± 0.56		1.35 ± 0.56	
Socioeconomic status ^***^	11.28 ± 2.76	11.64 ± 2.83	11.91 ± 2.77	12.01 ± 2.89	11.92 ± 2.85	12.36 ± 3.03		12.64 ± 2.94	
Social communication^*^	4.39 ± 1.81	4.43 ± 1.83	4.39 ± 1.77	4.40 ± 1.78	4.45 ± 1.78	4.48 ± 1.78		4.57 ± 1.76	
Social integration ^***^	8.70 ± 0.93	8.79 ± 0.94	8.91 ± 0.88	8.92 ± 0.92	8.89 ± 1.00	9.01 ± 0.96		9.11 ± 0.98	

Note: ^*^ *p* < 0.05, ^***^ *p* < 0.001. Analysis of variance (ANOVA) are used to examine the differences between cities

#### Social integration and the Gini coefficient

**Fig. 1:**
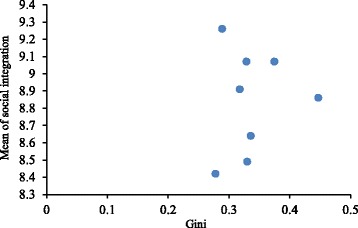
Scatter Plot of Mean of Social Integration with Gini Coefficient

Social integration showed significant differences between distinct levels of Gini (*p* < 0.001). Figure 1 shows that the relationship between Gini and social integration was non-linear. For example, the Gini of Jiaxing and Chengdu were less than 0.3, but the social integration of Jiaxing was lowest, and Chengdu’s social integration was highest. Therefore, it indicates that social integration is not monotonically related to absolute income inequality.

### Health

#### Health

IMs’ self-reported health, Subjective well-being and mental health was best in Beijing and lowest in Xiamen. Perception of stress was highest in Zhengzhou and lowest in Beijing (Table 3). The “Wilkinson hypothesis” (increased income inequality in a society is related to worse health performance [41]) was observed in self-reported health, but not in other health statuses. Spearman rank correlation between Gini coefficients and self-reported health (RS = −0.12, *P* < 0.001), subjective well-being (RS = −0.02, *P* < 0.001), and mental health (RS = −0.04, *P* < 0.001) showed significant inverse correlation, with perception of stress (RS = 0.09, *P* < 0.001) being an exception to that.

**Table 3:** Self-Reported Health, Subjective Well-Being, Perception of Stress and Mental Health of Eight Selected Cities in China

	0.2 < Gini ≤0.3		0.3 < Gini ≤0.4					0.4 < Gini ≤0.5	
	Jiaxing	Chengdu	Zhengzhou	Qingdao	Xiamen	Zhongshan	Beijing	Shenzhen	Total
Self-report health^***^	23.96 ± 3.66	23.32 ± 3.71	22.31 ± 3.86	23.5 ± 3.91	22.26 ± 3.8	23.19 ± 4.01	24.03 ± 3.77	23.16 ± 3.81	23.22 ± 3.87
Subjective well-being^***^	21.65 ± 5.76	21.97 ± 6.26	21.53 ± 5.86	20.89 ± 6.11	20.87 ± 6.42	22.43 ± 6.66	23.12 ± 6.32	22.3 ± 6.22	21.80 ± 2.65
Perception of stress^***^	9.12 ± 2.53	8.99 ± 2.68	10.04 ± 2.49	9.61 ± 2.58	10.00 ± 2.54	8.93 ± 2.72	8.69 ± 2.68	9.00 ± 2.52	9.30 ± 2.64
Mental health^***^	26.91 ± 2.81	27.02 ± 2.92	26.43 ± 3.07	26.25 ± 2.94	25.82 ± 3.57	26.55 ± 3.34	27.11 ± 2.89	26.53 ± 2.74	26.58 ± 3.07

Note: ^***^ *p* < 0.001. Analysis of variance (ANOVA) are used to examine the differences between cities

#### Relationship between health, social integration, and social income inequality

Elder IMs were worse in self-reported health (*P* < 0.001), but had less perceived stress (*P* < 0.01) and better subjective well-being (*P* < 0.01) and mental health (*P* < 0.05) than younger IMs. Men were better in self-reported health, but worse in subjective well-being. Interestingly, education had a negative relationship with self-reported health (*P* < 0.001), subjective well-being (*P* < 0.05), and mental health (*P* < 0.01), which we explain further in the discussion. IMs from rural districts were worse in self-reported health and had more perceived stress than those who came from urban districts, and IMs with longer years of residence had less perceived stress (*P* < 0.05). Social insurance could alleviate perceived stress (*P* < 0.05). Social communication was conducive to mental health (*P* < 0.01). Socioeconomic status, acculturation, and social integration willingness had a mostly positive relationship with four aspects of health status. There was an interaction effect between the Gini coefficient and socioeconomic status. Social income inequality had a negative effect on the health status of IMs by affecting their economic inclusion. With the same socioeconomic status, IMs living in cities with higher social income inequality experienced worse self-reported health, subjective well-being, and mental health. In addition, social income inequality and social communication interactively influenced mental health in a negative way, i.e., with the same level of social communication, more social income inequality had a negative relationship with mental health (Table 4).

**Table 4:** Coefficients of Linear Mixed Models of the Relationship between Health and Social Integration

	Self-reported health		Subjective well-being		Perception of stress		Mental health	
	β	Std. error	β	Std. error	β	Std. error	β	Std. error
Fixed effect								
Age	−0.432	0.036^***^	0.371	0.058***	−0.083	0.025**	0.073	0.030*
Gender	0.448	0.059^***^	−0.452	0.092***	−0.011	0.040	0.007	0.0474
Education	−0.236	0.068^***^	−0.261	0.105*	−0.012	0.046	−0.205	0.054***
Category of Hukou	0.396	0.092^***^	0.070	0.143	−0.169	0.062**	0.171	0.073*
Marital status	−0.275	0.07^***^	0.225	0.114*	−0.012	0.049	0.004	0.059
Years of residence	0.007	0.032	0.028	0.049	−0.051	0.022*	0.034	0.026
Social insurance	−0.018	0.195	0.586	0.303	−0.334	0.131*	−0.100	0.155
Gini × Social insurance	0.012	0.199	−0.561	0.309	0.244	0.134	0.163	0.159
Social communication	0.194	0.222	−0.344	0.345	−0.085	0.150	0.548	0.177**
Gini × Social communication	−0.178	0.224	0.425	0.348	0.123	0.151	−0.794	0.179***
Socioeconomic status	1.267	0.120***	3.586	0.311***	−1.173	0.136***	0.854	0.159***
Gini × Socioeconomic status	−0.683	0.231**	−1.756	0.359***	0.696	0.157***	−0.406	0.184*
Acculturation and integration will	0.324	0.121*	−0.074	0.167	−0.351	0.105**	0.218	0.088*
Gini × Acculturation and integration will	−0.029	0.209	0.766	0.285**	0.180	0.183	0.284	0.151
Random effect								
Gini	0.226	0.134	0.256	0.158	0.387	0.317	0.083	0.052

Note: Gender, education, category of Hukou, and marital status were binary classification variables (0,1,0 was reference), other variables were continuous and standardized in the models. *p < 0.05; ** *p* < 0.01; ***p < 0.001

## Discussion

This study examined the relationships between social income inequality, social integration, and health status of IMs in China. Extensive research suggests that social integration and economic inequality are determinants of a population’s health state [42–44], while little is known about the mechanisms governing how these two determinants produce health capital. This study modeled social integration with multi-dimensional measures and examined the interaction effects of social integration and social income inequality on IMs’ health status in China. Our findings not only supported a correlation between social integration and health status, but also found that social income inequality interacted with economic inclusion of IMs, and therefore was related to IMs’ heath status.

This study makes four key contributions to the literature on IMs’ social integration and health status.

First, we used multi-dimensional social integration indices to examine the relationship between social integration and IMs’ health status. We described social integration in four aspects: *socioeconomic status*, *integration willingness*, *social insurance*, and *social communication*. To begin with, *socioeconomic status*, including objective income and subjective economic feeling, was the most significant factor associated with internal migrants’ health status, but IMs tended to be in lower social economic status than residents because of institutional exclusion and deficiency in social capital [32].

Then, we found that the *integration willingness* had a significant correlation on self-reported health, perception of stress, and mental health. The result was consistent with the research found in other IM populations. For example, Garnweidner et al. (2012) showed that views on food habits and acculturation affected health among female immigrants [45, 46]. Chae et al. (2009) suggested that discrimination perception may be a risk factor for physical pain and self-rated health among American Indians/Alaska Natives [47]. Brayley et al. (2010) showed that social identity was associated with psychological constructs [48]. Social integration may be associated with psychological functioning, which includes a sense of belonging, personal control, and generalized trust [49].

Further, our results suggest that improved *social insurance* in IMs’ resident cities may help reduce their stress level. Due to the wide coverage of medical insurance across regions, social insurance might be the least important factor in social integration to influence IMs’ health, but how to transfer insurance coverage across regions for IMs is still uncertain. Since social health insurance has not been integrated at the national level, some IMs may not receive their customary social insurance if they have left their covered provinces [50]. Therefore, any improved social insurance in IMs’ resident cities may ensure the necessary access to care among IMs.

Our study supported the positive relationship between *social communication* and individual well-being that has long been recognized in the existing literature [51, 52], Nonetheless, limited evidence points to how social communication influences individual health status in migrants [53]. In summary, using four aspects of social integration (*socioeconomic status*, *integration willingness*, *social insurance*, and *social communication)* helps explain how social integration is related to health.

Our second contribution demonstrated that IMs’ social integration was not monotonically related to absolute income inequality. This challenges the traditional view of the negative relationship between social integration and absolute income inequality among non-IM populations. For example, Kawachi et al. (1997) found that income inequality was strongly correlated with lack of social trust, and in turn, both social trust and group membership were associated with total mortality [54]. Huisman et al. (2009) demonstrated that income inequality would have a relatively strong association with mortality after adjusting social capital indicators [42]. Weaver et al. (2006) showed that income inequality was moderately related to mortality and social capital had a powerful, negative effect on age-adjusted mortality rates [55]. More research is needed to explain non-monotonic relationships between absolute income inequality and social integration of IMs in China.

Third, we found that interaction between income inequality and social communication had a negative relationship with mental health. For example, in cities with higher income inequality, improved social communication may not benefit mental health [56]. IMs in China mostly joined the fellow-townsman associations, followed by participation in community sports groups. However, given the high social income inequality, these social communications may not provide adequate support to improve IMs’ mental health. Further research is needed to identify effective interventions to create more inclusive social capital and promote better mental health [57].

Finally, this study suggested that social income inequality may affect IMs’ health status through social integration, i.e., economic integration including objective economic income or subjective social feelings. More social income inequality had a negative impact on self-reported health, perceived stress, subjective well-being, and mental health, even at the same level of socioeconomic integration. Findings from previous studies have suggested that socioeconomic factors were a primary driver of health inequities [58], in accordance with our results.

It is worth noting that the IMs with less education had better self-reported health and mental health, which seems contradictory to the existing literature [59]. However, in China, IMs are a special population,most of whom have migrated from rural villages and had low educational attainment. For example, in our study, only 4.8% of IMs had a degree above high school. In general, if non-residents have college degrees or above, they can receive resident status via employment, i.e., some degree of naturalization, which means they are no longer IMs. Therefore, IMs with higher educational attainment were those small groups who cannot achieve resident status through employment. The selection bias of IMs with higher education may explain the inverse relationship between education and health among IMs. Future research is needed to examine the disparity in health among non-resident IMs or naturalized residents with high educational attainment.

This study has merely provided a snapshot of social inequality, social integration, and health among IMs in China, and there is no causality between the influencing factors and health status. We will extend the scope of the research in the future by examining more macro-social factors (e.g., policy and social culture) and grouping IMs in terms of residence time, education, and gender. In addition, we plan to compare the IMs with the registered population using qualitative studies, such as focus groups or in-depth interviews to complement the limitations of the quantitative studies. For example, the monthly income was used to calculate the Gini coefficient, which may underestimate socioeconomic inequality. Due to the cross-sectional design of the survey, we cannot establish the causality between social income inequality and health status among IMs in China.

## Conclusion

In conclusion, health status was associated with social integration and social income equality. Our findings suggest that young, female, and more-educated IMs may need more attention with regard to social integration. Policies or programs targeting IMs should be in support of integration willingness, sense of belonging, and economy. The role of the community should be expanded, such as creating community-based promotions for IMs. For example, social or sports activities are tailored to integration willingness and cultural adaption [60]. Meantime, social activities to facilitate employment and creating generalized trust within social environments should be promoted. Finally, macro-level structural changes and policies are needed to promote income equity.

## Appendix 1

**Table 5:** Internal Migrants’ Social Integration in 2014

Variables	Definition	Options	% or ^−^x ± s
Monthly household income	Income of all family members in the current residence		3500-7000^a^
Income, occupation position, and degree of respect compared with relatives, friends and colleagues at current residence	Subjective social status and level of respect compared to other people, measured by marking a “social ladder” (1 as the bottom status to 10 as the top status)		5.47 ± 1.60
			5.98 ± 1.60
Old-age insurance	Own either township workers’ old-age insurance, urban residents’ old-age insurance, or new rural social pension insurance	no	27.7
		yes	72.3
Medical insurance	Own either social medical insurance or commercial medical insurance	no	14.8
		yes	85.2
Number of organizations participated in (0–8)	Number of organizations respondents participated in, including labor union, volunteer associations, the Chinese Communist Party group of migrants/local residents, alumni association, chamber of commerce of hometown, fellow-townsman associations, and other organizations		0.40 ± 0.77
Number of activities attended (0–7)	Number of activities respondents attended, for example, community sports, social public welfare activities, election campaigns, awards events, the home owners’ committee, management activities of residents’ committees, and other activities		0.66 ± 1.04
Type of neighbors	Whether neighbors of respondents were registered residents, whohad “Hukou”, or migrants	Outsiders	43.5
		The locals	20.6
		Outsiders and locals	29.5
		Not sure	6.4
Consent of the views (8–40)	Those views include 7 problems about social norms or customs: 1) The customs of the hometown (such as the customs of marriage, funerals) is more important to you; 2) Working in the current place is more important to you than living in the hometown; 3) Your child should learn to speak the hometown dialect; 4) Maintaining the hometown’s lifestyle, such as eating habits, is important; 5) There is a big difference in health habits between you and local residents; 6) There is a big discrepancy in clothing, education, or retirement style between you and local residents; 7) Your opinions of some social issues are very distinct from the local residents’. Respondents provided their agreement with these views based on a five-point scale (strongly agree, agree, neither agree or not, disagree, strongly disagree).		23.80 ± 4.09
Familiarity with local dialect	Proficiency in the local language	Don’t understand	14.9
		Understand some only	23.0
		Understand and speak some	22.7
		Understand and speak	39.4
Integration will (9–36)	Consists of 9 questions, such as, “I would like to live together with locals in a block (community)”, “I would like to be a colleague with locals”, and “I would like to be a neighbor with locals”. Respondents answered based on a four-point scale (1 = disagree completely to 4 = agree completely), with the higher score meaning better integration will.		30.43 ± 4.28
Discrimination perception (4–16)	Includes 4 questions: “I feel the locals are willing to accept me as a part of them,” “I feel the locals don’t want to be my neighbors,” “I feel the locals don’t like me,” and “I feel the locals look down on me” (1 = disagree completely to 4 = agree completely), with higher score meaning more discrimination perception.		7.34 ± 2.16
Willingness to bring family members to local residence	Whether to bring the subject’s spouse, unmarried children, or parents to local residence in the next 1 to 3 years	All of family members at location	26.6
		Yes	23.6
		Yes, but only some	14.1
		No	30.7
		Not sure	4.9

Note: ^a^ interquartile

## Appendix 2

**Table 6:** Result of Rotated Component Matrix in social integration of internal migrant

	Component					
	1	2	3	4	5	6
	Factor one (acculturation and integration will)	Factor two (social insurance)	Factor three (economy)		Factor four (social communication)	
% of Total variance explained	12.6	11.9	20.6		20.1	
X1 The main of neighbors	0.005	−0.028	−0.008	0.028	−0.047	0.771
X2 Familiarity with the local dialect	0.123	0.090	0.022	−0.003	0.111	0.706
X3 Bring family members or not to the locale in the next 1–3 years	0.174	0.027	−0.048	0.758	−0.029	−0.016
X4 Income or occupation position compared with the relatives, friends, and colleagues in the current residence	0.038	0.017	0.869	0.055	0.034	0.024
X5 Degree of respect compared with relatives, friends, and colleagues in the current residence	0.137	0.032	0.847	0.063	0.037	−0.022
X6 The consent of the views	0.526	0.000	−0.071	0.022	0.012	0.309
X7 Integration will	0.789	0.039	0.135	0.052	0.057	0.017
X8 Discrimination perception	0.812	−0.035	−0.111	−0.024	−0.033	0.039
X9 Endowment insurance	0.031	0.866	0.021	0.059	0.116	0.036
X10 Medical insurance	0.036	0.882	0.028	−0.019	0.029	0.027
X11 Number of organizations participated in	−0.008	0.041	0.061	0.019	0.839	0.025
X12 Number of activities attended	0.098	0.097	0.007	−0.009	0.819	0.040
X13 Monthly household income	−0.085	0.009	0.162	0.742	0.039	0.046

## Abbreviations

IM: Internal Migrant

## References

[1] Lin Y, Zhang Q, Chen W, Shi J, Han S, Song X, Xu Y, Ling L (2016). Association between social integration and health among internal migrants in ZhongShan, China. PLoS One.

[2] Chen J (2011). Internal migration and health: re-examining the healthy migrant phenomenon in China. Soc Sci Med.

[3] Qiu P, Caine E, Yang Y, Chen Q, Li J, Ma X (2011). Depression and associated factors in internal migrant workers in China. J Affect Disord.

[4] Gui Y, Berry JW, Zheng Y (2012). Migrant worker acculturation in China. Int J Int Relat.

[5] Newbold B (2005). Health status and health care of immigrants in Canada: a longitudinal analysis. J Health Serv Res Policy.

[6] Newbold KB (2005). Self-rated health within the Canadian immigrant population: risk and the healthy immigrant effect. Soc Sci Med.

[7] Chen J, Chen S, Landry PF (2013). Migration, environmental hazards, and health outcomes in China. Soc Sci Med.

[8] Alvarez-Ude CF (2008). Rebollo AP: [psychological disturbances and deterioration of health-related quality of life of patients with stage 3-5 chronic kidney disease (not on dialysis)]. Nefrologia.

[9] Tong Y, Piotrowski M (2012). Migration and health selectivity in the context of internal migration in China, 1997-2009. Popul Res Policy Rev.

[10] Salant T, Lauderdale DS (2003). Measuring culture: a critical review of acculturation and health in Asian immigrant populations. Soc Sci Med.

[11] Li X, Stanton B, Chen X, Hong Y, Fang X, Lin D, Mao R, Wang J (2006). Health indicators and geographic mobility among young rural-to-urban migrants in China. World Health Popul.

[12] Li L, Wang HM, Ye XJ, Jiang MM, Lou QY, Hesketh T (2007). The mental health status of Chinese rural-urban migrant workers: comparison with permanent urban and rural dwellers. Soc Psychiatry Psychiatr Epidemiol.

[13] Wong DF, He X, Leung G, Lau Y, Chang Y (2008). Mental health of migrant workers in China: prevalence and correlates. Soc Psychiatry Psychiatr Epidemiol.

[14] Lau JT, Cheng Y, Gu J, Zhou R, Yu C, Holroyd E, Yeung NC (2012). Suicides in a mega-size factory in China: poor mental health among young migrant workers in China. Occup Environ Med.

[15] Gao J, Weaver SR, Fua H, Pan Z (2014). Does workplace social capital associate with hazardous drinking among Chinese rural-urban migrant workers?. PLoS One.

[16] Hilmers A, Bernabe-Ortiz A, Gilman RH, McDermott AY, Smeeth L, Miranda JJ. Rural-to-urban migration: socioeconomic status but not acculturation was associated with overweight/obesity risk. J Immigr Minor Health.2016;18(3):644–51. 10.1007/s10903-015-0234-9 PMC4861745 26087715

[17] Lin D, Li X, Wang B, Hong Y, Fang X, Qin X, Stanton B (2011). Discrimination, perceived social inequity, and mental health among rural-to-urban migrants in China. Community Ment Health J.

[18] Xing H, Yu W, Chen S, Zhang D, Tan R (2013). Influence of social support on health-related quality of life in new-generation migrant Workers in Eastern China. Iran J Public Health.

[19] Zhao S, Chen X, Wang L (2015). Maternal parenting and social, school, and psychological adjustment of migrant children in urban China. Int J Behav Dev.

[20] Worku EB, Woldesenbet SA (2015). Poverty and inequality - but of what - as social determinants of health in Africa?. Afr Health Sci.

[21] Biggs B, King L, Basu S, Stuckler D (2010). Is wealthier always healthier? The impact of national income level, inequality, and poverty on public health in Latin America. Soc Sci Med.

[22] Wilkinson RG, Pickett KE (2006). Income inequality and population health: a review and explanation of the evidence. Soc Sci Med.

[23] Jin J, Wang J, Ma X, Wang Y, Li R (2015). Equality of medical health resource allocation in China based on the Gini coefficient method. Iran J Public Health.

[24] Liu Y, Jiang Y, Tang S, Qiu J, Zhong X, Wang Y (2015). Analysis of the equity of emergency medical services: a cross-sectional survey in Chongqing city. Int J Equity Health.

[25] Yang J. From isolation, selection into the integration: thinking of theory of social integration of internal migrants. Popul Stud. 2009;33(1):17–29.

[26] Rose R (2000). How much does social capital add to individual health? A survey study of Russians. Soc Sci Med.

[27] Hyyppa MT, Maki J (2001). Why do Swedish-speaking Finns have longer active life? An area for social capital research. Health Promot Int.

[28] Yang J. Index of assimilation for rural-to-urban migrants: a further analysis of the conceptual framework of assimilation theory. Popul Econ. 2010;2:64–70.

[29] Butler M, Warfa N, Khatib Y, Bhui K (2015). Migration and common mental disorder: an improvement in mental health over time?. Int Rev Psychiatry.

[30] Haasen C, Demiralay C, Reimer J (2008). Acculturation and mental distress among Russian and Iranian migrants in Germany. Eur Psychiatry.

[31] Jin L, Wen M, Fan JX, Wang G (2012). Trans-local ties, local ties and psychological well-being among rural-to-urban migrants in shanghai. Soc Sci Med.

[32] Wen M, Fan J, Jin L, Wang G (2010). Neighborhood effects on health among migrants and natives in shanghai, China. Health Place.

[33] Lisa F, Berkman TG, Brissette I, Teresa E. Seeman: From social integration to health: Durkheim in the new millennium. Soc Sci Med. 2000;51(6):843–57. 10.1016/s0277-9536(00)00065-4 10972429

[34] Chen R, Zhao Y, Du J, Wu T, Huang Y, Guo A (2014). Health workforce equity in urban community health service of China. PLoS One.

[35] Nong N (2014). Interpretation of the Gini coefficient algorithm. Popul Sci Technol.

[36] Zhou H. Measurement and theoretical perspectives of immigrant assimilation in china. Popul Res. 2012;36(3):27–37.

[37] Newnham EA, Harwood KE, Page AC (2007). Evaluating the clinical significance of responses by psychiatric inpatients to the mental health subscales of the SF-36. J Affect Disord.

[38] Diener E, Emmons RA, Larsen RJ, Griffin S (1985). The satisfaction with life scale. J Pers Assess.

[39] Karam F, Berard A, Sheehy O, Huneau MC, Briggs G, Chambers C, Einarson A, Johnson D, Kao K, Koren G (2012). Reliability and validity of the 4-item perceived stress scale among pregnant women: results from the OTIS antidepressants study. Res Nurs Health.

[40] Kim G, DeCoster J, Bryant AN, Ford KL. Measurement equivalence of the K6 scale:the effects of race/ethnicity and language. Assessment. 2015;23(6);555–68. 10.1177/1073191115599639 PMC5468522 26282779

[41] Bakkeli NZ (2016). Income inequality and health in China: a panel data analysis. Soc Sci Med.

[42] Huisman M, Oldehinkel AJ (2009). Income inequality, social capital and self-inflicted injury and violence-related mortality. J Epidemiol Community Health.

[43] Tsai AC, Lucas M, Sania A, Kim D, Kawachi I (2014). Social integration and suicide mortality among men: 24-year cohort study of U.S. health professionals. Ann Intern Med.

[44] Baumgartner JN, Susser E (2013). Social integration in global mental health: what is it and how can it be measured?. Epidemiol Psychiatr Sci.

[45] Vue W, Wolff C, Goto K (2011). Hmong food helps us remember who we are: perspectives of food culture and health among Hmong women with young children. J Nutr Educ Behav.

[46] Garnweidner LM, Terragni L, Pettersen KS, Mosdol A (2012). Perceptions of the host country's food culture among female immigrants from Africa and Asia: aspects relevant for cultural sensitivity in nutrition communication. J Nutr Educ Behav.

[47] Chae DH, Walters KL (2009). Racial discrimination and racial identity attitudes in relation to self-rated health and physical pain and impairment among two-spirit American Indians/Alaska natives. Am J Public Health.

[48] Brayley N, Obst PL (2010). The Australian Grey nomads--are they who we think they are? Enhancing formative research through the quantitative assessment of psychological constructs. Health Promot J Austr.

[49] Cattell V (2001). Poor people, poor places, and poor health: the mediating role of social networks and social capital. Soc Sci Med.

[50] Wang Q, Zhang D, Hou Z (2016). Insurance coverage and socioeconomic differences in patient choice between private and public health care providers in China. Soc Sci Med.

[51] Marcus AF, Echeverria SE, Holland BK, Abraido-Lanza AF, Passannante MR (2016). The joint contribution of neighborhood poverty and social integration to mortality risk in the United States. Ann Epidemiol.

[52] Seeman T. Social ties and health: the benefits of social integration. Soc Ties Health. 1996:442–51. 10.1016/s1047-2797(96)00095-6 8915476

[53] Chen H, Zhu Z, Sun D, Wang X. The Physical and Psychological Health of Migrants in Guangzhou, China: How Does Neighborhood Matter?. Inquiry A Journal of Medical Care Organization Provision & Financing. 2016;53:1–8. 10.1177/0046958016668065 PMC5798746 27637270

[54] Kawachi KB, Lochner K, Prothrow-Stith D. Social capital, income inequality, and mortality. Am J Public Health. 1997:1491–8. 10.2105/ajph.87.9.1491 PMC1380975 9314802

[55] Weaver RR, Rivello R (2006). The distribution of mortality in the United States: the effects of income (inequality), social capital, and race. Omega (Westport).

[56] Jorge-Monteiro MF, Ornelas JH (2016). What's wrong with the seed? A Comparative Examination of an Empowering Community-Centered Approach to Recovery in Community Mental Health. Community Ment Health J.

[57] Na L, Hample D (2016). Psychological pathways from social integration to health: an examination of different demographic groups in Canada. Soc Sci Med.

[58] Boen C (2016). The role of socioeconomic factors in black-white health inequities across the life course: point-in-time measures, long-term exposures, and differential health returns. Soc Sci Med.

[59] Dalgard OS, Mykletun A, Rognerud M, Johansen R, Zahl PH (2007). Education, sense of mastery and mental health: results from a nation wide health monitoring study in Norway. BMC Psychiatry.

[60] Tsay SF, Hsu YN, Chen SF, Shen SH, Lin HY (2015). A community-based experience model of mental-social health promotion for older people in Taichung City. Hu Li Za Zhi.

